# Use of RNA sequencing to evaluate rheumatic disease patients

**DOI:** 10.1186/s13075-015-0677-3

**Published:** 2015-07-01

**Authors:** Eugenia G Giannopoulou, Olivier Elemento, Lionel B Ivashkiv

**Affiliations:** Biological Sciences Department, New York City College of Technology, City University of New York, New York, NY 11201 USA; Arthritis and Tissue Degeneration Program and the David Z Rosensweig Genomics Research Center, Hospital for Special Surgery, New York, NY 10021 USA; HRH Prince Alwaleed Bin Talal Bin Abdulaziz Alsaud Institute for Computational Biomedicine and Department of Physiology and Biophysics, Weill Cornell Medical College, New York, NY 10021 USA

## Abstract

Studying the factors that control gene expression is of substantial importance for rheumatic diseases with poorly understood etiopathogenesis. In the past, gene expression microarrays have been used to measure transcript abundance on a genome-wide scale in a particular cell, tissue or organ. Microarray analysis has led to gene signatures that differentiate rheumatic diseases, and stages of a disease, as well as response to treatments. Nowadays, however, with the advent of next-generation sequencing methods, massive parallel sequencing of RNA tends to be the technology of choice for gene expression profiling, due to several advantages over microarrays, as well as for the detection of non-coding transcripts and alternative splicing events. In this review, we describe how RNA sequencing enables unbiased interrogation of the abundance and complexity of the transcriptome, and present a typical experimental workflow and bioinformatics tools that are often used for RNA sequencing analysis. We also discuss different uses of this next-generation sequencing technology to evaluate rheumatic disease patients and investigate the pathogenesis of rheumatic diseases such as rheumatoid arthritis, systemic lupus erythematosus, juvenile idiopathic arthritis and Sjögren’s syndrome.

## Introduction

Gene expression profiling is the analysis of the expression or activity of genes, in order to understand how genes contribute to certain biological functions, and to elucidate the pathogenic mechanisms of diseases and disorders. In order to acquire new information about the role of genes under various conditions, gene expression is typically measured in different conditions or tissues, such as at different time points, or between normal and cancer/disease cells. Differential expression (DE) analysis between conditions produces gene signatures that are characteristic of the condition or disease being studied. For example, a gene signature in rheumatoid arthritis (RA) is a collection of genes that are either up- or down-regulated when compared with normal cells (for example, monocytes). In the field of rheumatic diseases, gene expression profiling studies have used DNA microarrays extensively [1–3]. DNA microarrays, introduced over 15 years ago, have been routinely used as a gene expression analysis approach that can measure transcript abundance on a genome-wide scale [4]. This technology relies on arrays of oligonucleotide probes that capture mRNA complementary sequences (cDNA) present in biological samples at various concentrations. Microarray assays are ideal for targeted identification of already known messenger RNAs. However, their limited coverage does not allow the detection of rare or novel transcripts, splice variants, or low-abundance transcripts. Microarrays have low sensitivity compared with other approaches (for example, real-time PCR), suffer from the problem of background hybridization, and have limited dynamic range that often prevents accurate assessment of low signal intensities.

Such limitations are largely absent in RNA sequencing (RNA-seq), a next-generation sequencing (NGS) method largely used for the genome-wide measurement of RNA abundance and the detection of alternative splicing events [5, 6]. Compared with microarrays, RNA-seq has several advantages, such as low background signal, since RNA sequence reads can often be unambiguously mapped to unique regions of the genome, increased sensitivity and high reproducibility between technical and biological replicates. RNA-seq is free from the probe-specific hybridization of microarrays, and has a broader dynamic range, allowing the unbiased detection of novel transcripts, both coding and noncoding. Examples of noncoding transcripts are: long (>200 bp) non-coding RNAs (lncRNAs) that are implicated in diverse biological processes, are critical for controlling cell state decisions in pluripotent cells, and may physically associate with chromatin proteins to regulate gene expression; enhancer RNA (eRNA), a class of relatively short non-coding RNA molecules transcribed from the DNA sequence of enhancer regions, whose transcription is positively correlated with the mRNA levels of the surrounding protein-coding genes; microRNA (miRNA), short non-coding RNAs (18 to 24 bp) that can cause silencing or degradation of mRNA, ultimately leading to a decrease in the amount of protein, with or without changes in the number of mRNA transcripts. It also requires relatively small amounts of input RNA and is suitable for detecting alternative spliced transcripts, alternative promoters and 3′ untranslated region usage, measuring allele-specific expression and detection of chimeric and fusion transcripts [6–9]. With the rapid advances in NGS technology, a more comprehensive and accurate RNA-seq-based transcriptome analysis has become feasible. Just like microarrays, design of RNA-seq experiments is important and key factors include number of replicates, sequencing depth, single-end or paired-end sequencing and more [6]. Finally, like all NGS-based experiments, RNA-seq produces a great amount of data the analysis and interpretation of which requires a significant computational infrastructure, as well as custom analytic pipelines and databases.

In this review, we present a typical RNA-seq workflow, experimental choices and data analysis pipelines. We also discuss recent published studies (Table 1), as well as related abstracts, showing the variety of uses of this NGS technology to study the transcriptome of patients with RA, systemic lupus erythematosus (SLE), juvenile idiopathic arthritis (JIA), and Sjögren’s syndrome (SS).

**Table 1:** Rheumatic disease studies using RNA-seq technology

Disease	Sample size	Cell type	RNA-seq application	Reference
JIA	3 JIA patients, 3 patients at clinical remission, 3 healthy controls	PBMCs	Non-coding RNA (lncRNAs)	[48]*
RA	2 RA patients, 2 healthy controls	RASFs	DE transcript/gene analysis	[44]
RA	6 RA patients	PBMCs	Biomarker discovery	[47]*
SLE	9 SLE patients, 8 healthy controls	Human monocytes	DE transcript/gene analysis	[45]
SLE	6 SLE patients, 3 healthy controls	PBMCs	Single gene profiling	[53]
SS	50 SS patients, 37 healthy controls	Whole blood cells	Non-coding RNA (lncRNAs)	[49]*
SS	6 SS patients, 3 healthy controls	Minor salivary glands	Non-coding RNA (miRNAs)	[50]

*Non-peer-reviewed abstracts. DE, differential expression; JIA, juvenile idiopathic arthritis; lncRNA, long non-coding RNA; PBMC, peripheral blood mononuclear cell; RA, rheumatoid arthritis; RASF, rheumatoid arthritis synovial fibroblast; RNA-seq, RNA sequencing; SLE, systemic lupus erythematosus; SS, Sjögren’s syndrome

## Experimental choices in RNA sequencing

In the past few years, sequencing technologies and chemistries have been advancing at a rapid pace. Several companies offer NGS platforms, with Illumina’s HiSeq and MiSeq [10], and Life Technologies’ Ion Torrent (Applied Biosystems) [11] being the leading platforms for RNA-seq. Each has its unique advantages and limitations; thorough overviews and comparisons between several NGS platforms are provided in [12–14].

**Fig. 1:**
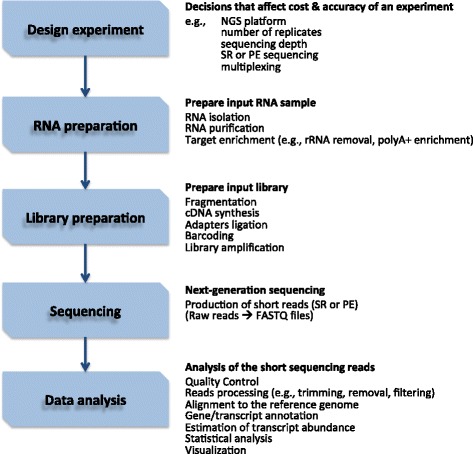
A typical RNA-seq workflow. RNA sequencing (RNA-seq) is a multi-step process that involves designing the experiment, preparing the RNA sample and the input library, using a next generation sequencing platform, and performing analysis on the short sequenced reads. NGS, next-generation sequencing; PE, paired-end; SR, single-read

Independently of the technology of choice, a typical RNA-seq workflow is a multi-step process that includes RNA and library preparation, sequencing, and data analysis (Fig. 1). During RNA preparation, the fraction of RNA to profile is isolated and purified and specific RNA classes are enriched, either by direct enrichment or depletion of other classes. There are several target enrichment methods, such as removal of rRNA or polyadenylated positive enrichment. Library preparation includes converting RNA to cDNA, cDNA fragmentation, attaching platform-specific adapter sequences at the ends of the cDNA fragments, and library amplification. Importantly, both RNA and library preparation choices depend on the sequencing platform used, as well as on the experimental objective. For example, different preparation libraries are required for the profiling of small RNA targets, such as miRNA (for example, RNA isolated through size-selection), nuclear RNA [15], and chromatin-associated total RNA [16]. The library is then sequenced on a NGS platform, producing millions of short sequence reads that correspond to one or both ends of the cDNA fragments, called single reads (SRs) and paired-end (PE) reads, respectively. The short reads are then aligned to the appropriate reference genome and analyzed using programs that are specific for RNA-seq data analysis and distinct from those used for microarray analysis.

Researchers face several challenges when designing an RNA-seq experiment regarding decisions that can affect both the cost and the accuracy of the experiment. Such challenges involve selecting the optimal number of replicates and the sequencing depth required to achieve reliable detection power of DE. As far as replicate choice is concerned, several studies [17–19], as well as the ENCODE RNA-seq standards [20], recommend performing experiments with two or more biological replicates (Zhang and colleagues [19] mention that the number of differentially expressed genes plateaus at approximately four replicates). Technical replicates from the same library are typically not necessary, due to the high technical reproducibility achieved by the sequencing technology [17]. The discussion regarding sequencing depth is more complicated since sequencing depth is highly dependent on the objectives of the RNA-seq study, genome size, and transcriptome complexity. For example, the ENCODE RNA-seq standards [20] suggest that transcriptome profiling of polyadenylated positive samples require modest depths of sequencing, such as 30 million PE reads of length >30 bp, while for novel transcript discovery and strong quantification of known transcript isoforms deeper sequencing is required. A study focusing on the sequencing depth of RNA-seq in chickens [21] suggests that 30 million SRs (75 bp) are needed to achieve reliable measurement of mRNA expression across all genes, while 10 million SRs (75 bp) allow the detection of approximately 80% of annotated chicken genes. However, a study by Liu and colleagues [22] suggests that 300 million PE reads (2 × 101 bp) are needed to detect approximately 80% of the differentially expressed genes from samples that derived from adipose of a healthy individual before and after systemic administration of endotoxin (lipopolysaccharide (LPS)), and at least 400 million PE reads are necessary to achieve approximately 80% detection rate of alternative splicing events. Although the optimal number of reads per sample cannot be easily determined without taking into account several factors of an experiment, most studies agree that a much higher sequencing depth is required for the identification of alternative splicing events compared with DE of genes, and that the depth of sequencing has more impact on low rather than on high abundance transcripts [22, 23]. Despite sequencing depth being an important consideration in RNA-seq, it has been shown that the number of biological replicates is a more significant factor than sequencing depth, or technical replicates, in the accurate detection of differentially expressed genes [18, 23, 24]. Thus, since budget is often a concern, it is recommended to increase the number of samples, which correspond to biological replicates, sequenced at a modest depth, rather than to increase sequencing depth in fewer samples.

Another decision that researchers have to make before sending their samples to a sequencing facility for library preparation is whether to choose SRs or PE reads. As mentioned above, SRs refer to fragments that are read by the sequencer from only one end to the other, generating the sequence of base pairs, while for PE reads the sequencer starts at one read end, finishes this direction at the specified read length and then starts another round of reading from the opposite end of the fragment. PE reads is a necessary configuration in an RNA-seq experiment when increased mapping accuracy is important in order to assess genome rearrangements, identify fusion genes and detect alternative splicing events. Although SRs are usually preferred as an RNA-seq strategy for measuring transcript abundance due to lower cost, it is recommended to perform PE sequencing whenever possible [25, 26].

Finally, multiplexing is a method that allows pooling libraries from multiple samples into a single sequencing reaction. In order to identify the ‘origin’ of sequenced reads, a short nucleotide sequence (approximately six to seven nucleotides), called the barcode or index, is attached to each cDNA fragment during library preparation. The barcodes are read during sequencing, allowing the reads to be traced back to their original samples. Choosing to perform RNA-seq multiplexing can reduce the cost of an experiment, but will also produce smaller number of reads per sample. For example, a single flow cell lane from an Illumina HiSeq 2500 platform routinely produces approximately 200 million SRs for one sample without multiplexing. If four samples are multiplexed, then approximately 50 million SRs per sample will be produced at a much lower sequencing cost per sample.

## Data analysis pipeline

From the data analysis perspective, RNA-seq includes the following steps: assessment of the quality of the sequenced reads (using tools such as FastQC [27]), removal or trimming low quality reads (using tools such as Trimmomatic, or Sickle), alignment (or mapping) of the remaining reads to the reference genome and annotation of transcripts to which reads have been mapped, estimation of transcript abundance, and statistical analysis to identify DE or splicing among samples. It is important to note that *de novo* assembly of RNA-seq data is also commonly used for studying the transcriptome of species without reference genomes, such as non-model organisms in microbiome and metagenomics studies; however, discussion of RNA-seq as a *de novo* transcript assembly tool is beyond the scope of this review.

Read alignment remains one of the most computationally intensive steps in the entire process, since it requires the alignment of tens or hundreds of millions of reads to multiple gigabases for a typical mammalian genome. Some of the most popular alignment programs used for RNA-seq include Bowtie/TopHat [25], BWA [28], and STAR [29]. Importantly, RNA-seq aligners need to identify reads that map across splice junctions. An evaluation of alignment algorithms in the RNA-seq context is described in [30].

The next step involves estimating the abundance of known genes or transcripts. Frequently, this involves determining the number of reads that map to known genes or transcripts, also known as read counts. In this analysis, the number of reads supporting each gene or transcript according to gene annotation (for example, RefSeq, ENSEMBL, UCSC Genes) are determined using programs such as HTSeq [31]. Although read counts are quantitative approximations of the abundance of target transcripts, these counts must be normalized to remove technical biases and parameters inherent in the preparation steps for RNA-seq, such as the length of the transcript and the sequencing depth. For example, deeper sequencing results in higher counts, biasing comparisons between different runs with different sequencing depths. Similarly, longer transcripts are more likely to have reads mapped to their region, resulting in higher counts, biasing comparisons between transcripts of different lengths. Fragments per kilobase of exon per million fragments mapped (FPKM) is a way to normalize read counts; programs such as Cufflinks [25] also estimate the absolute expression levels of genes/transcripts in FPKM values. Cufflinks and related programs use intelligent strategies to assign reads to transcripts when multiple and partially overlapping transcript isoforms are present. FPKM values provide user-friendly gene and transcript level quantifications, suitable for creating heatmap visualizations and comparing expression between genes. It is important to mention that FPKMs correspond to PE RNA-seq experiments that produce two reads per fragment, while RPKM values (reads per kilobase of exon per million fragments mapped) are used when a SR RNA-seq strategy is applied. Cuffdiff [25], which is part of Cufflinks, finds differentially expressed genes and transcripts in more than one condition and tests for significant differences.

However, it has been discussed in some studies that RPKMs/FPKMs have certain limitations that can bias estimates of DE [24, 32] and may not be an appropriate way to normalize RNA-seq reads. Thus, read counts are typically used as input to programs like DESeq [33], EdgeR [34] and limma (voom) [35], which are amongst the most commonly used and freely available DE software packages. These programs perform non-FPKM normalization of read counts (for example, using LOWESS regression, or quantile normalization), estimate read count fold changes between conditions at the gene or the transcript level, and assess the statistical significance of observed read count differences. Statistical significance analysis also includes correction for multiple testing, often in the form of false discovery rate control. It is also frequently used in conjunction with minimum fold-change requirements (for example, 2-fold, 10-fold) so as to ensure biological relevance. A comprehensive evaluation of several DE analysis methods for RNA-seq data can be found in [24].

For the identification of non-coding RNA, such as miRNA and lncRNA, the data analysis pipelines differ from the ones used for DE analysis of genes/transcripts. After the reads are aligned against the reference genome, non-coding annotations are used, such as the miRBase (database of known miRNAs), lncRNAdb (database of lncRNAs), ncRNAdb (database of non-coding regulatory RNAs) and others. Related third party analysis tools for this purpose include mirRanalyzer [36], miRTools [37], and lncRScan [38]. Similarly, for estimating the expression of diploid organisms at the haplotype, isoform and gene levels, specific tools are needed to be part of the RNA-seq pipeline, such as MMSEQ [39].

Visualization of the mapped reads (that is, either raw reads or read densities) in a genome browser, such as the UCSC Genome Browser [40] or the Integrative Genomics Viewer [41], is a common step in the RNA-seq data analysis pipeline. This genome-wide display of reads facilitates the exploration of RNA-seq datasets, as well as hypothesis generation, sharing and integration with other genomic data, such as published ENCODE tracks. However, visualization cannot quantify expression levels nor find global patterns; the steps that were described before provide the systematic genome-wide quantification of information in RNA-seq experiments. Combinations of the tools mentioned above, together with general bioinformatics tools like R/Bioconductor and Galaxy, can create different RNA-seq pipelines adapted to the needs of each project.

## Identifying dysregulated pathways in disease cells

RA is a chronic systemic autoimmune disorder that primarily affects the joints and ultimately leads to their destruction [42]. It affects approximately 1% of the general population and is characterized by functional disability, and increased morbidity and mortality, mainly due to accelerated atherosclerosis. RA synovial fibroblasts (RASFs) play a vital role in the initiation and prolongation of RA, due to the production of cytokines, chemokines, and matrix-degrading enzymes, which lead to the thickening of the joint membrane, and progressive destruction of cartilage and bone [43]. The characterization of cytokine signaling pathways involved in RA provides an opportunity for the identification of pro-inflammatory cytokines that can be targeted for novel RA therapy. A recent study [44] describes the use of RNA-seq to profile the RASF transcriptome in order to gain insight into the roles of synovial fibroblasts (SFs) in RA. The study reveals a complete picture of differentially expressed genes and their isoforms in RASFs, and provides a global transcriptional insight into the novel roles of synovial SFs in the pathogenesis of RA. RNA-seq was performed on samples from RASF-derived RNA of two adult female RA patients and from SF RNA of two healthy female donors; the latter were used as normal controls. A mean value of approximately 84 million reads per sample was obtained, and DE was estimated on the gene and transcript levels, as well as alternative promoter usage and alternative splicing. The ratio of the RA group to the control group was estimated for every gene/transcript along with the statistical significance of differences between the values, and two categories of differential gene/isoform expression were identified. The first one consists of genes/isoforms expressed uniquely in control SFs or only in RASFs, while the second category consists of genes/isoforms with at least two-fold up-regulated or down-regulated expression between control SFs and RASFs.

In this study [44], several genes and isoforms, not previously associated with RA, were identified: 214 genes were found uniquely expressed in SFs and 682 genes were only expressed in RASFs; 122 and 155 genes were up- and down-regulated, respectively, by at least two-fold in RASFs compared with SFs; 343 known and 561 novel isoforms were up-regulated and 262 known and 520 novel isoforms were down-regulated by at least two-fold. Within the top differentially expressed genes, the authors identified genes that have been reported previously to be associated with RA. Importantly, the magnitude of difference and the number of differentially expressed known and novel gene isoforms were all significantly higher than achieved previously by DNA microarrays. Network and pathway analysis performed on the differentially expressed genes and their known isoforms revealed strong representation of inflammatory response and cell death. Although these pathways have been predicted previously to correlate with RA, this study provides a more complete list of genes/isoforms involved in these pathways. Besides known inflammatory and immune responses, other novel dysregulated networks, such as cell morphology, cell-to-cell signaling and interaction, cellular movement, cellular growth and proliferation, cellular development, antigen presentation pathway, atherosclerosis signaling, LXR/RXR activation, and role of *BRCA1* in DNA damage response, were found to potentially contribute to the pathogenesis of RA. Overall, this study shows the first complete transcriptome analysis of SFs from patients with RA using RNA-seq and reveals a complete repertoire of active molecules, networks and pathways of differentially expressed genes and their isoforms in RASFs. As suggested by the authors, follow-up analyses using a larger number of patient samples will be necessary to validate the alterations in transcriptional regulation reported in this study and provide the resources necessary to elucidate the molecular mechanisms underlying the role of SFs in the pathogenesis of RA.

The study by Shi and colleagues [45] used RNA-seq to perform a whole transcriptome analysis of patients with SLE and compare gene expression with that of healthy controls. SLE is considered to be the quintessential systemic autoimmune disease. Gene expression studies of peripheral blood mononuclear cells (PBMCs) from patients with SLE have demonstrated a type I interferon (IFN) signature and increased expression of inflammatory cytokine genes. Although SLE is characterized by elevated type I IFN production, the underlying etiopathogenesis of SLE remains obscure, particularly at the level of dysregulated gene expression. RNA-seq was used to perform a comprehensive transcriptome analysis of primary human monocytes from eight healthy controls and nine SLE patients, with no evidence of other autoimmunity. The results of this study are numerous and focus not only on the altered expression of coding and non-coding transcripts, but also on a thorough qualitative characterization of the monocyte transcriptome of SLE patients. First, among known protein-coding genes, there was evidence of global repression with a large number of known protein coding genes expressed in normal monocytes, but silenced in SLE. These genes were highly enriched with processes related to embryo development, suggesting that SLE monocytes are more differentiated. Second, many down-regulated genes in SLE monocytes were also related to cell proliferation and cell adhesion, while up-regulated genes were related to active inflammation, immune response and cytokine activity. Third, it is reported that SLE patients had diminished expression of most endogenous retroviruses and small nucleolar RNAs, but exhibited increased expression of pri-miRNAs. Moreover, some novel loci expressed at higher abundance in SLE monocytes were inducible by LPS, known to activate type I IFNs. Although the authors of that study did not perform extensive validation of the classes of these novel transcripts found to have altered expression, we believe that they could be eRNAs, whose expression may correlate with mRNA levels of nearby genes, suggesting the potential regulatory and functional role of these SLE-specific regions. LPS and microbial products have also been demonstrated to accelerate renal disease and induce lupus-like processes in mice. This finding provides an additional perspective from which to understand SLE. Importantly, this study also revealed increased circulating LPS, which induces type I IFN expression, in SLE patients. The authors examined the concordance of coding genes expressed in SLE, after stimulation with LPS and after stimulation with alpha-IFN, and found considerable overlap, demonstrating that endotoxin can, in part, mimic the type I IFN signature seen in SLE. Whether endotoxin could represent a biomarker for disease severity, as well as how nucleic acid-driven toll-like receptors TLR7, TLR8 and TLR9 could be implicated, remains to be determined.

Overall, this study [45] showed that monocytes from SLE patients exhibit globally dysregulated gene expression. The transcriptome is not simply altered by the transcriptional activation of a set of genes, but is qualitatively different in SLE. The identification of novel transcripts, inducible by LPS, suggests that chronic microbial translocation could contribute to the immunologic dysregulation in SLE, a new potential disease mechanism. Finally, the importance of this study lies in the identification of multiple features of altered transcription and processing in SLE, which potentially contribute to the pathologic processes of this still enigmatic disease.

## RNA-seq for biomarker discovery

Abatacept (CTLA4Ig) belongs to the biologic class of drugs, which means that it works similarly to natural substances in the immune system and is used to decrease inflammation in RA [46]. Although abatacept generally improves outcomes for RA patients, up to 40 to 50% of RA patients fail to respond to the drug. The identification of potential biomarkers that can predict abatacept responsiveness is the goal of the study by Henkel and colleagues [47]. Although this study is briefly described in a non-peer-reviewed abstract and uses only six subjects, it showed that RNA-seq-based transcriptome analysis of PBMCs of six RA patients treated with abatacept may elucidate mechanistic and biomarker-related pathways altered in PBMCs by drug therapy. RNA samples were derived from PBMCs from six RA patients treated with abatacept with or without oral disease-modifying antirheumatic drugs and with or without prednisone. Five of these patients were positive for anti-CCP antibodies (markers for diagnosis and prognosis in RA), while all six patients had active disease at baseline despite recent tumor necrosis factor inhibitor therapy (based on the mean DAS28-CRP RA score; DAS28-CRP is a quantitative measure of RA where values >5.1 indicate high activity of the disease, <3.2 low activity of the disease and <2.6 remission). Two groups of patients were found according to DAS28-CRP scores at baseline and at 6 months after abatacept initiation. The responders group consists of three of the RA patients, while the other three RA patients belong to the non-responders group. PBMC RNA samples from all six patients were sequenced prior to receiving abatacept and approximately 2 months after abatacept initiation. DE analysis identified genes that (1) differed at baseline between abatacept responders and non-responders, and (2) changed between baseline and 2 months for both groups of responders and non-responders. A larger proportion of transcripts were significantly differentially expressed from baseline to 2 months in the responders group (6,339 transcripts) compared with non-responders (117 transcripts), while there was relatively little overlap between the differentially expressed genes of the responders and non-responders from baseline to 2 months (<10 transcripts). The authors then focused on the expression of genes related to T- and B-cell functions to identify baseline predictors of response (that is, genes significantly different at baseline between responder and non-responder groups) and 2-month predictors of response (that is, genes significantly different between 2 months and baseline). Interestingly, RNA transcripts for IgG isotypes and *IL-17* were reported as 2-month predictors of a 6-month clinical response, although their baseline levels of transcripts did not predict efficacy. In contrast, *IL6R* was a good baseline predictor of efficacy but its expression did not change from baseline to 2 months. The results that are briefly presented in this study [47] demonstrate the potential of RNA-seq as an assay for monitoring responses to drug therapies, such as abatacept, in PBMCs from RA patients.

## Identification of non-coding RNA

A recent study [48] that is briefly described in a non-peer-reviewed abstract used RNA-seq to identify differentially expressed protein-coding and non-coding transcripts in three JIA patients with active disease, three patients at clinical remission, and three healthy controls. JIA, also known as juvenile rheumatoid arthritis, is the most common rheumatic disease of childhood, and the goal of this study is to shed light on the genetic etiology and pathogenesis of this disease. RNA-seq was used on RNA samples isolated from PBMCs. DE analysis (≥1.2-fold) revealed 119 differentially expressed genes in active disease compared with control, 83 differentially expressed genes in the active disease compared with clinical remission condition, and 19 differentially expressed in clinical remission compared with control. Differentially expressed genes in active disease versus control and in active disease versus clinical remission were associated with connective tissue disorders, immunological disease and inflammatory disease (for example, CCR5, IL3RA and IL8). Interestingly though, among the non-protein coding transcripts, the authors observed DE in active disease versus control of two lncRNAs at chromosomal location 10p12.1 (*P* = 0.001, fold change = −3.73 and −4.74) and one lncRNA at 5q33.3 (*P* = 0.023, fold change = 3.99), with yet unclear biological functions. Overall, the authors of this abstract used RNA-seq to create gene signatures of different disease states in JIA, but also to detect novel lncRNAs that may have functional consequences in JIA.

The study described in [49] (also a non-peer-reviewed abstract) used RNA-seq to characterize SS patients, evaluating both coding and non-coding transcripts. SS is a common, clinically heterogeneous autoimmune disorder mainly affecting exocrine glands that disrupts tear and saliva secretion, leading to symptoms of dry mouth and eyes. RNA-seq was performed on samples that were isolated from whole blood of 57 SS patients and 37 healthy controls. DE analysis was performed and a total of 2,614 differentially expressed transcripts were identified. *SRP14*, *UQCRB* and *ATP5I* were the most statistically differentially expressed protein-coding transcripts between SS and control. Further investigation is required to study the biological functions of these genes and their potential role in SS. DE analysis of non-coding transcripts revealed a lncRNA at 2p25.1, a region found to be associated with transcription factor binding sites. This RNA-seq study [49] of SS patients identified candidate loci and differentially expressed lncRNA regions. Despite the function of these lncRNAs being unknown at the moment, future studies in SS are required to elucidate their functional effects.

Apart from lncRNAs, miRNAs have also been studied with RNA-seq in SS patients. Tandon and colleagues [50] used RNA-seq to characterize miRNAs in minor salivary glands of SS patients and healthy volunteers, with focus on the identification and discovery of novel miRNA sequences that may play a role in the disease. Although SS etiology is complex, with environmental, genetic, and genomic factors contributing, recently miRNAs have been investigated as potential diagnostic biomarkers in SS [51]. Total RNA was isolated from minor salivary glands of six patients with SS and three healthy volunteers. Sequenced reads that were not mapped to known human miRNAs from miRBase, nor to the human transcriptome, were used for novel miRNA predictions by miRanalyzer. A total of 15 novel miRNA candidates were predicted from this study. Using the RNAs from individual patients, six of these previously unidentified miRNAs were validated by quantitative PCR (that is, hsa-miR-4524b-3p, hsa-miR-4524b-5p, hsa-miR-5571-3p, hsa-miR-5571-5p, hsa-miR-5100, and hsa-miR-5572). The authors also tested for the presence of these miRNAs in other cell types and found all six miRNAs amplified in the Jurkat T (that is, immortalized T lymphocyte) and HSG (that is, immortalized human salivary gland) cell types. Interestingly, one of the validated novel miRNAs (hsa-miR-5100) was amplified by quantitative PCR in all samples, was differentially expressed between patients and healthy volunteers, and increased drastically as salivary flow was decreasing. According to miRBase, a very similar sequence (two mismatches) was found in mouse B cells (mmu-miR-5100). Since all patients selected for this study had low lymphocytic infiltration, the authors suggest that hsa-miR-5100 increase is possibly correlated with salivary dysfunction rather than with an increase in B cells. Although this study [50] used RNA-seq to sequence the transcriptome of six SS patients for the discovery of novel miRNAs, follow-up studies on a larger cohort of patients are required to validate the disease specificity and potential of this miRNA as a candidate prognostic marker for SS, as well as to characterize other miRNAs that correlate with the functional status of the salivary gland.

## Profiling of gene-specific splicing

Interferon regulatory factor 5 (IRF5) is a transcription factor that regulates the expression of pro-inflammatory cytokines and type I IFNs and is believed to be involved in the pathogenesis of SLE. Genetic variants of the *IRF5* gene have been associated with susceptibility to SLE in multiple populations; in each population, a distinct group of *IRF5* single nucleotide polymorphisms and genetic variants form haplotypes that confer risk for, or protection from, the development of SLE. It has been demonstrated that IRF5 expression is up-regulated in primary purified PBMCs from SLE patients and that up-regulation associates with IRF5-SLE risk haplotype monocytes [52]. It has been shown that alternative splicing of *IRF5* is elevated in SLE patients, as well as that human *IRF5* exists as multiple alternatively spliced transcripts with distinct function. Stone and colleagues [53] used RNA-seq to explore whether SLE patients express a unique IRF5 transcript signature compared with healthy donors, and whether an IRF5-SLE risk haplotype can define the profile of *IRF5* transcripts expressed.

Using standard molecular cloning techniques, the authors first identified and isolated 14 new differentially spliced *IRF5* transcript variants from purified monocytes of three healthy donors and six SLE patients. RNA-seq was subsequently used in order to obtain a more accurate and in-depth estimate of the differences between IRF5 transcript expression in primary immune cells of healthy donors and SLE patients. The most important finding of this study is that RNA-seq results (analyzed with MMSEQ) correlated with cloning and gave similar abundance rankings in SLE patients. This indicates the power of RNA-seq to identify and quantify spliced transcripts of a single gene at a greater depth compared with molecular cloning. Moreover, the authors of the study provide evidence that SLE patients express a different *IRF5* transcript signature from healthy donors and that the IRF5-SLE risk haplotype is among the top four most abundant *IRF5* transcripts expressed in SLE patients. Finally, this study suggests that RNA-seq of mammalian transcriptomes can provide a wealth of information on transcript assembly and abundance estimates and, because of its unbiased nature, it can be useful for *de novo* junction discovery.

## Conclusion

High-throughput NGS has marked the new age of biomedical research, since it offers the ability to sequence entire genomes or transcriptomes within days and to mine for previously unknown sequences in an unbiased manner. NGS methods have already been used to study a variety of biological systems and have been valuable tools in identifying markers for activity and progression in a variety of diseases. The advantages of RNA-seq in particular allow us to illustrate and study the complexity of transcriptomes more comprehensively.

In this review, we present studies based on the RNA-seq transcriptome analysis of patients with RA, SLE and SS that aim to shed light on the mechanisms of these rheumatic diseases. Importantly, we show the variety of RNA-seq applications and their flexibility to provide both quantitative and qualitative characterization of the transcriptomes under study. Gene expression profiling of RA patients was used to study the role of SFs in the pathogenesis of the disease, and gene signatures of the monocyte transcriptome in SLE patients showed globally dysregulated gene expression. Novel lncRNAs were identified from PBMCs of patients with JIA, as well as from SS patients, but further studies are required to elucidate whether these have functional consequences in these diseases. The discovery of novel miRNAs and disease biomarkers from minor salivary glands of patients with SS was also feasible with RNA-seq. In-depth single gene profiling was achieved by RNA-seq, revealing an *IRF5* transcript signature of SLE patients that is distinct from healthy donors and an IRF5-SLE risk haplotype in the top four most abundant *IRF5* transcripts expressed in SLE patients and not in healthy donors. Although these studies involved only a small number of patient samples, they all show the potential of RNA-seq as a tool to assess and study different rheumatic diseases.

As already discussed, the advantages and applications of RNA-seq are multifold. To our understanding, the main challenges of RNA-seq originate from the large amounts of data generated and involve the computational complexities associated with data analysis. As RNA-seq is becoming more affordable for research labs, the only daunting challenge is to select the most appropriate programs and tools for a specific RNA-seq application and to be able to understand and control the algorithmic parameters. Aside from these informatics challenges, which are steadily being overcome as more user-friendly and fast programs become available, RNA-seq is a particularly advantageous technology that embraces the complexity of the transcriptome and provides a mechanism to understand the underlying regulatory code.

## Note

This article is part of the series ‘*New technologies*’. Other articles in this series can be found at [54].

## Abbreviations

bp: base pair
DE: differential expression
eRNA: enhancer RNA
FPKM: fragments per kilobase of exon per million fragments mapped
IFN: interferon
IL: interleukin
JIA: juvenile idiopathic arthritis
lncRNA: long non-coding RNA
LPS: lipopolysaccharide
miRNA: microRNA
NGS: next-generation sequencing
PBMC: peripheral blood mononuclear cell
PE: paired-end
RA: rheumatoid arthritis
RASF: rheumatoid arthritis synovial fibroblast
RNA-seq: RNA sequencing
RPKM: reads per kilobase of exon per million fragments mapped
SF: synovial fibroblast
SLE: systemic lupus erythematosus
SR: single-read
SS: Sjögren’s syndrome
